# Quarantine as filter: a conceptual framework from early modern Venice

**DOI:** 10.1093/emph/eoag016

**Published:** 2026-07-19

**Authors:** Andrea Vianello, Robert H Tykot, Swamy R Adapa, Gloria C Ferreira, Gregory O'Corry-Crowe, Rays H Y Jiang

**Affiliations:** Department of Anthropology, University of South Florida, Tampa, FL USA; Department of Anthropology, University of South Florida, Tampa, FL USA; USF Genomics, Global Health Infectious Disease Research Center (GHIDR), Global Health, College of Public Health, University of South Florida, Tampa FL USA; Department of Molecular Medicine, Morsani College of Medicine, University of South Florida, Tampa, FL, USA; Department of Chemistry, College of Arts and Sciences, University of South Florida, Tampa, FL, USA; Hadrbor Branch Oceanographic Institute, Florida Atlantic University, Fort Pierce, FL, USA; Department of Molecular Medicine, Morsani College of Medicine, University of South Florida, Tampa, FL, USA

**Keywords:** quarantine, archaeology, plague, ancient DNA, pathogen evolution, Venice

## Abstract

Lazzaretto Vecchio, founded in 1423 in the Venetian lagoon, is recognized as the earliest permanent quarantine hospital. For nearly three centuries it operated at a major maritime frontier. Historical records describe extensive epidemic mortality and mandate burial on quarantine islands, leading to the long-standing model of a Venetian plague house dominated by resident burials (H1). Yet the island’s physical capacity is insufficient to accommodate all documented epidemic deaths, exposing a persistent discrepancy between archaeological evidence and early modern narratives of epidemic containment. Here, we synthesize historical, archaeological, and demographic evidence and argue that the available evidence does not support the interpretation of Lazzaretto Vecchio as a comprehensive cemetery for Venice’s epidemic dead. Instead, the burial assemblage is most consistent with selective demographic filtering associated with quarantine practice. We present two complementary explanatory models: (H2A) the Ellis Island Effect, dominated by intercepted travelers; and (H2B) the Secret Republic Fire, in which peak urban mortality was diverted through undocumented crisis disposal. Together, H2A and H2B describe a dual-regime containment system most consistent with the available evidence. By reframing quarantine as an active filtering process rather than a passive repository of epidemic mortality, this perspective highlights how governance and infrastructure shaped the burial record and created a unique opportunity to reconstruct infectious disease diversity and evolutionary trajectories across three centuries.

## INTRODUCTION AND CURRENT UNDERSTANDING OF VENETIAN QUARANTINE BURIALS

**Figure 1:**
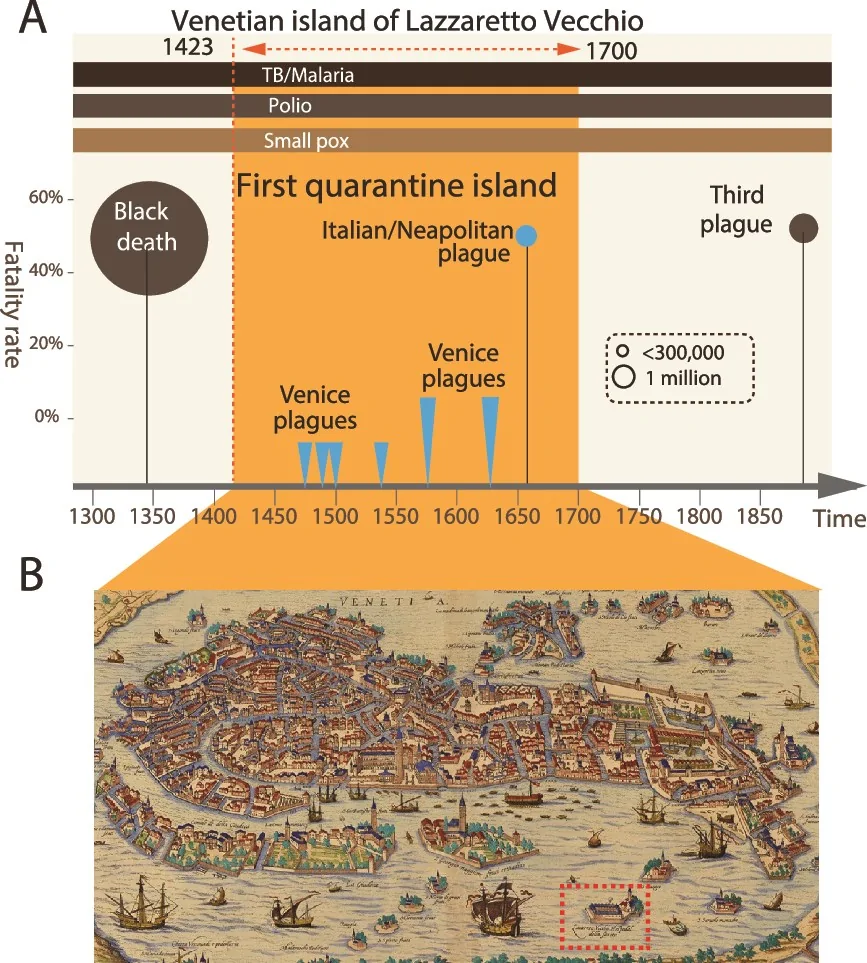
Venetian quarantine timeline and early cartographic context. (A) Timeline of major infectious diseases and key historical events spanning the ~300-year operation of the Venetian quarantine system. Circle size indicates the approximate relative mortality burden of each epidemic (count from historical sources). Blue triangles mark major Venetian plague outbreaks on the timeline. TB, tuberculosis. (B) *Lazzaretto Vecchio* depicted on the 1572 *Venetia* map by Georg Braun and Frans Hogenberg (*Civitates Orbis Terrarum*). Located southeast of the city center, the island is outlined here by a red dashed box, illustrating its established role within Venice’s maritime, quarantine, and public health infrastructure by the 16th century.

For over three centuries, the republic of Venice implemented a system of quarantine (Fig. 1A). At the heart of this system stood Lazzaretto Vecchio, an artificial island designated in 1423 as the first permanent quarantine hospital (*lazaretto*) for maritime travelers arriving with suspected infectious diseases [1–4] (Fig. 1B). A second island, Lazzaretto Nuovo, was established ~50 years later to accommodate and separate asymptomatic individuals from severe cases [5, 6]. This quarantine island system was unique in Europe for its sustained use and its dedicated role in infectious disease management, in contrast to most plague sites, which primarily reflect single-outbreak burial events [7–12]. Venice built a physical and bureaucratic firewall against contagion, sequestering ships, goods, animals, and people within a regulated space for 40 days (*quarantena*) to interrupt transmission before it reached the city’s densely populated core [2, 13].

The Venetian Republic maintained one of Europe’s earliest formal public health bureaucracies, the *Provveditori alla Sanità* (Health Magistracy), established in 1486 and endowed with policing, surveillance, and burial authority [4]. These magistrates directed maritime cordons, inspected ships and goods, issued “sanitary passes,” and ordered the confinement and burial of suspected plague victims, including Venetian residents, on the quarantine islands [4]. The Venetian system appears to have been effective in later centuries [3, 14]: it dampened the impact of the Neapolitan plague of 1656–1658 and suffered no major mass-fatality plagues thereafter, unlike Marseille or London [15, 16].

Lazzaretto Vecchio operated from 1423 to approximately 1700 as Venice’s primary quarantine island for the severely ill. In theory, the lazaretti should hold the physical remnants of centuries of outbreak management [4, 17, 18], particularly during the catastrophic epidemics of 1576 and 1630 [13]. This expectation is reinforced by historical records. During the devastating plague of 1576 [13, 19], Venice lost an estimated 50 000 lives, nearly one third of its population, and again in 1630 [20], ~46 000 died in a single year, despite strict quarantine enforcement.

Archaeological investigations conducted by Italian authorities since the 1980s have identified over 100 burial features of varying size [5, 6], including larger pits (containing up to 81 individuals) and smaller tomb-like contexts (typically fewer than five individuals), although only a limited number of large pits have been fully excavated and characterized. While ongoing studies continue to refine the site record, modern construction of administrative buildings and a museum limits the extent of complete excavation. To date, excavations have yielded approximately 1559 individuals [5, 21, 22].

The current history-based interpretation of Venetian quarantine burials in the literature assumes that Lazzaretto Vecchio functioned as a comprehensive burial ground for infectious disease mortality, containing a mixture of local residents and travelers. Because resident deaths would have constituted the majority of epidemic mortality, this model predicts a burial assemblage dominated by local inhabitants. We refer to this conventional interpretation (H1) as the Venetian Plague House model.

In this study, we show that this conventional model is difficult to reconcile with the available evidence. Specifically, we identify three key observations: (1) the limited physical burial capacity of the island relative to documented epidemic mortality, (2) eyewitness accounts from contemporary Venetian sources describing alternative crisis-period disposal practices, and (3) the demographic composition of the excavated assemblage, particularly the marked scarcity of children. Together, these observations do not support the conventional interpretation (H1) of Lazzaretto Vecchio and motivate two alternative models that better explain the archaeological and historical evidence.

## THE MISSING MORTALITY PARADOX: EVIDENCE AGAINST A COMPREHENSIVE CEMETERY MODEL

The physical burial capacity of Lazzaretto Vecchio was inherently constrained by its small size and shallow depth as an artificial island. The 1559 individuals excavated to date, representing perhaps 5%–15% of the island’s total burial capacity [5, 23], and the total capacity of the two lazaretti [17, 24, 25] account for about 10% of all infectious-disease deaths recorded for Venice during the 3 centuries [21, 22]. If most plague and infectious deaths documented in Venetian archives, likely numbering in the hundreds of thousands [13, 19], had been buried on the islands as mandated, the cumulative volume would have exceeded the physical capacity of this small artificial island many times over.

The temporal pattern reveals the paradox. Typical crisis cemeteries such as East Smithfield in London or La Corderie in Marseille were filled within months, each preserving the demographic cross-section of a single epidemic wave [10, 15, 26]. In contrast, Venice’s quarantine island operated continuously for nearly three centuries, receiving both travelers and residents who died under isolation. If the island had functioned as the primary burial ground for Venice’s epidemic mortality, as assumed under the conventional model (H1), its limited burial capacity would likely have been exhausted within a relatively short period. Yet the archaeological record instead reveals modest burial density accumulated over centuries.

There is also a striking lack of archaeological evidence for large-scale crisis plague burial documented elsewhere [27–30]. For example, the well-studied East Smithfield cemetery in London, a purpose-built Black Death burial ground, contained long mass-burial trenches with hundreds of individuals stacked in multiple layers in two large trenches, and is estimated to have accommodated approximately 2400 plague victims in total [10]. In contrast, the Venetian site displays only small burial trenches, modest numbers of interments [5, 6], and no clear stratigraphic evidence of a high-mortality mass event. If Lazzaretto Vecchio functioned as the primary burial ground for Venice’s epidemic dead, repeated large-scale mortality events would be expected to have produced comparable large crisis-burial deposits. Their absence therefore represents an important inconsistency with the conventional Venetian Plague House model (H1).

Our central argument is that current archaeological and demographic evidence does not support the conventional interpretation of Lazzaretto Vecchio as a comprehensive cemetery for Venetian epidemic mortality. Instead, the burial assemblage is most consistent with selective demographic and pathogen filtering associated with quarantine practice.

This reinterpretation reframes the burial assemblage not simply as a record of epidemic mortality, but as a selectively preserved archive of intercepted human hosts, pathogens, and disrupted transmission chains. As such, it provides a unique opportunity to reconstruct infectious disease diversity, transmission dynamics, and evolutionary history across nearly three centuries of quarantine operation.

## RE-EXAMINING MULTIPLE LINES OF EVIDENCE

The conventional interpretation of Lazzaretto Vecchio assumes that it functioned as a comprehensive burial ground for all infectious disease mortality. Re-evaluating this interpretation requires consideration of several dimensions of quarantine practice that are often conflated in the existing literature [2, 4, 13, 19, 23]: (i) function, as both a control point for incoming travelers and a site for resident quarantine; (ii) utility, as a combined medical facility and burial ground for infectious disease deaths; (iii) temporal dynamics, encompassing routine operation versus peak outbreak management; and (iv) the divergence between archival directives and actual practice, where official records emphasize universal quarantine and burial, while real-world implementation remains uncertain. These dimensions are critical because they determine who entered quarantine, who was ultimately buried on the island, and which pathogens were preserved within the burial assemblage.

### Evidence of historical eyewitness accounts: Rocco Benedetti (1576)

Direct testimony from the Venetian chronicler Rocco Benedetti provides rare, explicit documentation of emergency plague management during the catastrophic outbreak of 1576 [31]. Writing in Venetian, the administrative language of the Republic, Benedetti offers an internal account of quarantine operations at Lazzaretto Vecchio, produced by observers embedded within Venice’s civic and public-health apparatus rather than by later commentators. (Full Benedetti testimony quotes are in supplemental texts).

Benedetti repeatedly describes large-scale burning of bodies, both on Lazzaretto Vecchio itself, where smoke and stench from cremation were constant, and, when conditions became intolerable, at auxiliary sites on the Lido, where bodies were layered with lime and soil in deep trenches and rapidly incinerated. These passages indicate that during peak mortality, burial was suspended and replaced by mass cremation [31]. As Benedetti records: “et si vedevano da tutte le hore nuvoli di fumo stendersi in aere largamente per l’abrusciar de corpi.” (“and at all times one could see clouds of smoke spreading through the air from the burning of bodies.”).

Although Benedetti’s testimony has been noted in historical sources [31, 32], it has not been incorporated into current interpretations of the quarantine burial record. We consider this account important because it demonstrates that crisis-period management differed from routine quarantine operations. Specifically, it provides direct evidence that large-scale cremation was employed during epidemic peaks, despite official mandates emphasizing burial and despite the absence of such practices in the conventional Venetian Plague House model (H1). The testimony therefore suggests that the relationship between mortality and burial was not constant through time and that emergency disposal practices may have selectively excluded a portion of peak epidemic mortality from the archaeological record.

Benedetti’s account supports the possibility that quarantine operated under distinct routine and crisis-phase regimes. This observation forms one of the key lines of evidence motivating the reinterpretation proposed in this study.

### Evidence from archaeology—burials structure and demographics

The burial data from Lazzaretto Vecchio suggest a pattern consistent with steady, long-term containment rather than catastrophic epidemic mortality. Across the excavated sectors of over a hundred features [6, 21, 22], most contain only 1–5 individuals, with a small subset of outliers containing up to 81 individuals [6]. The overall frequency distribution of burial counts per feature reflects stochastic accumulation over an extended duration, rather than abrupt mass interment. This burial pattern is unexpected for a plague crisis site. In true epidemic emergencies [27–30], we typically observe sharp spikes in burial rates, and dense mass-deposition layers associated with overwhelming mortality.

Crucially, no archaeological signatures of acute hasty deposition have been identified: there is no evidence of spatial compression of remains. Excavation challenges must be acknowledged, only three large pits have been fully characterized archaeologically out of 108 recognized features, due to extensive modern construction [6]. Although only three large pits have been fully excavated, these represent distinct areas and the full range of burial morphologies observed on the island, providing a meaningful, albeit partial, window into mortuary practice.

Where pits were recovered intact, bodies were found to be treated with lime and orderly stacked between 1 and 5 layers [6], and observations suggest deposition before rigor mortis, indicating rapid handling of the corpses per Venetian public health protocol rather than unmanaged prolonged storage [6].

The total excavated minimum number of individuals is 1559 [6]. This number is far lower than historical expectations for major epidemic peaks in Venice. If the Lazzaretto had handled 50 or more burials per day during peak crises for even a few years, the cemetery would have been rapidly overwhelmed. The absence of such accumulation therefore points to the interpretation that the Lazzaretto Vecchio was not the primary burial ground for Venice’s local peak epidemic dead, but instead a controlled quarantine cemetery with limited throughput.

Even more striking is the demographic profile. Children, who typically represent a large share of deaths in plague pits elsewhere, are scarce. At canonical Black Death and later plague cemeteries such as Marseille and Martigues, children constitute approximately 24%–29% of burials [33], and can reach up to 46% when adolescents are included [12], reflecting household transmission and generalized mortality during uncontrolled outbreaks. In Italy specifically, contemporary witnesses describe overwhelming pediatric mortality, i.e. “Plague of children.” Ranieri di Sardo in Pisa reported 80% mortality among children aged 12 or younger during the 1374 outbreak, and similar patterns were observed in Siena [34].

By contrast, Lazzaretto Vecchio exhibits a pronounced adult bias. Based on archival demographics, 32%–37% of the Venetian population at the time were children, and mortality among them was historically high [35]. Thus, at least one-third of burials would be expected to be non-adults if the site reflected the full spectrum of urban epidemic mortality. Yet, the original archaeological report recorded only 17.1% children/subadults (238 of 1390 individuals) [6]. Within the small-sized burial contexts specifically, only 14.2% (24 of 169) were children/subadults [6]. Notably, the original demographic assessments were conducted during archaeological excavation rather than through dedicated forensic anthropological reanalysis. In the current combined collections under archaeological and anthropological curation, representation is even lower: fewer than 50 children are present among ~1300 individuals curated at the University of South Florida (USF), Florida, USA, and in Bologna, Italy, representing less than 5% of the assemblage. This marked deficit of children is difficult to reconcile with a burial population dominated by local epidemic mortality and household transmission.

Taken together, the archaeological evidence reveals three recurring patterns: steady burial accumulation, limited burial density, and a strongly adult-biased demographic composition. These observations are difficult to reconcile with the conventional Venetian Plague House model (H1), which predicts a burial assemblage dominated by local epidemic mortality and household demographics. Instead, the archaeological evidence is more consistent with a selectively filtered burial population, as expected under the traveler-dominated and crisis-diversion models proposed in this study.

## REINTERPRETING VENETIAN QUARANTINE: A DUAL-REGIME SYSTEM

**Figure 2:**
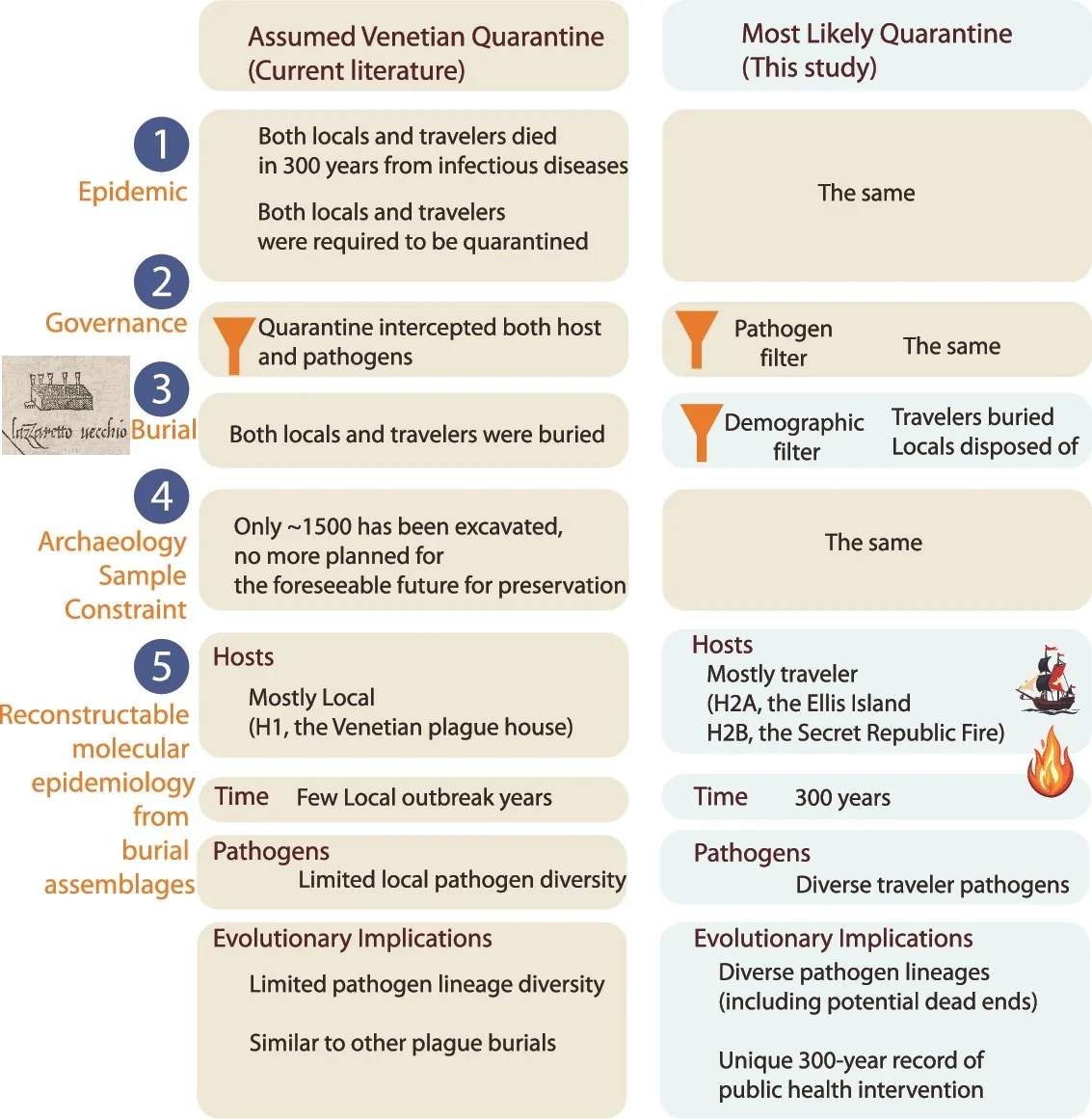
Five-layer model of Venetian quarantine and reconstructed epidemic signals. The figure contrasts the traditionally assumed model of Venetian quarantine (left; current literature) with the reconstructed model proposed in this study (right). Layer 1 (Epidemic) represents the true mortality landscape over ~300 years, in which both local residents and travelers died from infectious diseases and were theoretically subject to quarantine. Layer 2 (Governance) illustrates institutional interception and containment, where quarantine functioned as a pathogen filter by selectively intercepting infected hosts, travelers, and transmission chains. Layer 3 (Burial representation; differs in our model) highlights the divergence between official mandates and operational reality. Under the traditional interpretation, both locals and travelers were buried on the lazaretto; in the reconstructed model, traveler-associated deaths were preferentially retained in the burial record, whereas many local epidemic deaths were likely excluded during crisis periods, creating a demographic filter. Layer 4 (Archaeological sampling constraint) emphasizes that only a limited portion of the site (~1500 individuals) has been excavated, with little additional excavation anticipated due to preservation constraints, thereby imposing a sampling limit on the observable record. Layer 5 (Reconstructable epidemics; differs in our model) summarizes the epidemiological signal preserved in the burial assemblage. Under the traditional “Venetian plague house” model (H1), the site would primarily reflect local outbreaks and a limited set of local pathogens. In contrast, the dual-regime model proposed here, combining the Ellis Island Effect (H2A) and the Secret Republic Fire (H2B), predicts a burial assemblage enriched for traveler-associated mortality and diverse intercepted pathogens accumulated over three centuries. Together, these sequential filters transform true mortality into a highly selective archaeological and molecular record. Current literature assumptions are shown in grey, whereas the aspects revised in this study are shaded in blue. Detail of the island of Lazzaretto Vecchio, from *Map of Venice* (L0064135), Wellcome Collection (2018-03-30), CC-BY-4.0.

The available historical, archaeological, and demographic evidence suggests that the burial assemblage represents a selectively filtered subset of mortality shaped by quarantine practice. We summarize this reinterpretation of the Venetian quarantine system, in contrast to conventional views, in the five-layer framework presented in Fig. 2, which compares true mortality, infectious disease interception, burial representation, archaeological sampling constraints, and the reconstructable molecular epidemiology preserved within the burial assemblage.

**Figure 3:**
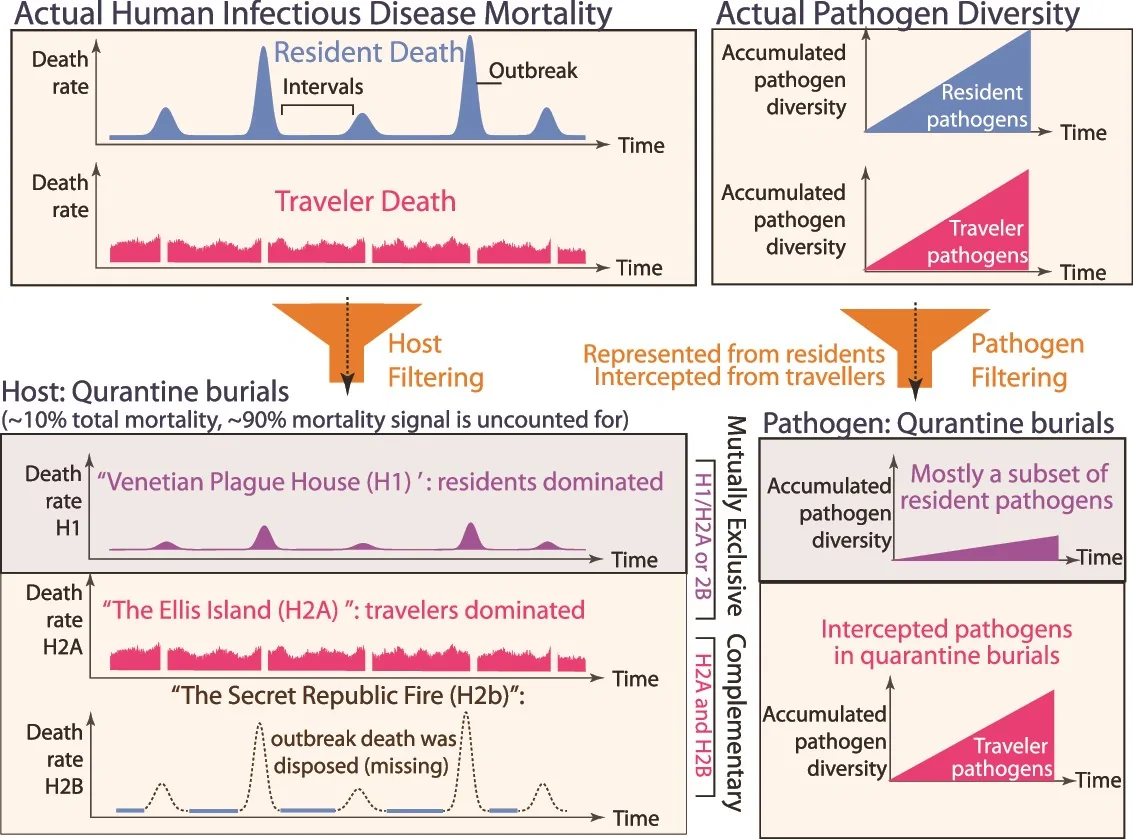
Quarantine filtering of human mortality and pathogen diversity at Lazzaretto Vecchio. Both human remains and pathogen diversity preserved at Lazzaretto Vecchio reflect the outcome of quarantine-based filtering processes. Schematic comparisons illustrate true mortality (upper panel) and pathogen diversity *versus* those captured in quarantine burials (lower panel). Total mortality comprises both resident and traveler deaths: resident mortality is characterized by episodic peaks separated by long intervals, whereas traveler mortality follows a relatively steady baseline. The burial record therefore represents a filtered subset of total mortality. Under a random sampling model (H1), burials would be dominated by residents and household demographics, and pathogen diversity would largely reflect local outbreak strains with limited diversity. In contrast, under a traveler-dominated intake (H2A), combined with the exclusion or diversion of peak resident mortality during crises (H2B), burials are expected to be dominated by non-resident individuals. In this case, pathogen diversity reflects intercepted infections at the port of entry, including lineages that failed to establish transmission, leading to higher cumulative diversity. Current evidence does not support H1 and is instead consistent with a combined H2A–H2B filtering regime. Axes represent relative death rate (left panels) and accumulated pathogen diversity (right panels) over time; values are schematic and intended to illustrate conceptual dynamics rather than quantitative measurements.

Figure 3 illustrates the central epidemiological logic of the competing models. Total mortality in Venice likely consisted of episodic resident outbreak peaks superimposed on a relatively steady background of traveler-associated mortality. Under the conventional Venetian Plague House model (H1), the burial assemblage would be expected to reflect local epidemic mortality, household demographic structure, and pathogen diversity dominated by resident outbreak strains. However, the limited burial density, pronounced adult bias, scarcity of children, and historical evidence for crisis-phase disposal practices are not readily explained by this interpretation.

In contrast, under the combined H2A–H2B model, traveler-associated deaths are preferentially retained whereas many peak-crisis local deaths are excluded from the burial record. The resulting assemblage is therefore enriched for non-resident individuals and diverse intercepted pathogen lineages, including infections that failed to establish sustained transmission. Accordingly, the available evidence is most consistent with a dual-regime quarantine system composed of two complementary operational modes: (1) a routine traveler-interception regime (H2A), and (2) a crisis-phase mortality diversion regime (H2B).

## THE ELLIS ISLAND EFFECT (H2A): ROUTINE TRAVELER INTERCEPTION

The archaeological evidence is consistent with a reinterpretation of Lazzaretto Vecchio as a quarantine filtering station for outsiders arriving at Venice’s maritime frontier rather than as a comprehensive civic plague cemetery. Analogous to a premodern “Ellis Island,” the lazaretto intercepted foreign travelers, sailors, merchants, and maritime laborers arriving from infected regions before they entered the city proper.

Under this framework, those interred at Lazzaretto Vecchio were disproportionately transient individuals caught at the intersection of global trade and quarantine enforcement. Venice’s containment strategy relied on geographic segregation, using the lagoon itself as a physical barrier separating citizens from imported contagion. The resulting burial assemblage therefore represents not a random sample of epidemic mortality, but a selectively filtered population shaped by maritime mobility and quarantine policy.

Several lines of archaeological evidence are consistent with this interpretation. Excavated burials are overwhelmingly adult, with a marked paucity of children. Burial features are predominantly small and low-density rather than catastrophic mass graves. Together, these patterns suggest a slowly accumulated mortality stream associated with regulated quarantine operations rather than generalized epidemic collapse.

This reinterpretation also generates testable predictions. If the burial assemblage is traveler-enriched, individuals should exhibit high isotopic heterogeneity, limited kinship clustering, and pathogen diversity reflecting repeated introductions from multiple geographic origins rather than sustained local transmission. Stable isotope analysis, ancient DNA, kinship reconstruction, and burial spatial modeling provide direct approaches for evaluating these expectations.

Taken together, the demographic composition, burial structure, and historical context are consistent with H2A and support the interpretation of Lazzaretto Vecchio as a selective demographic and pathogen filter operating at the maritime frontier.

## THE SECRET REPUBLIC FIRE (H2B): CRISIS-PHASE MORTALITY DIVERSION

Whereas H2A describes routine quarantine operations, H2B addresses extreme epidemic conditions during catastrophic mortality surges. For most of its operational history, Lazzaretto Vecchio likely processed a limited and highly regulated mortality stream. However, during major outbreaks such as 1576 and 1630, the system appears to have operated under fundamentally different conditions.

Historical testimony from Rocco Benedetti describes large-scale burning of bodies at both Lazzaretto Vecchio and auxiliary sites on the Lido during the 1576 plague. Combined with the archaeological absence of high-density mass burial deposits and the limited physical burial capacity of the island, these accounts suggest that emergency disposal practices operated alongside or replaced routine burial procedures during crisis periods.

Large-scale cremation represents one plausible mechanisms consistent with the available evidence. It accommodates the logistical constraints of the Venetian lagoon, the discrepancy between documented mortality and burial capacity, and the absence of corresponding mass-fatality deposits. Alternative explanations, such as sea disposal or large-scale return of infected bodies to the city, conflict with Venetian maritime priorities and quarantine policies emphasizing containment and rapid sanitation.

Importantly, H2B is structurally inferred rather than directly reconstructed. We do not propose a detailed crisis-disposal system, but instead identify a minimal explanatory framework consistent with the historical testimony, archaeological observations, and logistical realities of managing epidemic mortality during extreme outbreaks.

This interpretation generates testable expectations. If crisis-period mortality was diverted from routine burial pathways, major epidemic peaks should be underrepresented in the burial assemblage relative to historical mortality records. High-resolution radiocarbon chronology, stratigraphic modeling, and pathogen phylogenetics may help determine whether major outbreak periods are disproportionately absent from the surviving burial record.

Taken together, the available historical and archaeological evidence is consistent with H2B and indicates that peak epidemic mortality was managed differently from routine quarantine deaths. This distinction between routine and crisis-phase operations provides a plausible explanation for the apparent mismatch between documented mortality and the surviving burial assemblage.

## THE MOST LIKELY SCENARIO: A DUAL-REGIME CONTAINMENT SYSTEM

H1, which assumes that the burial assemblage represents a relatively unbiased sample of Venetian epidemic mortality, is not supported by the available archaeological, demographic, and historical evidence. Instead, current evidence is most consistent with a combined H2A–H2B regime.

Under this interpretation, Lazzaretto Vecchio functioned both as a quarantine filter intercepting infected travelers during routine operations and as a crisis-management system in which peak urban mortality was diverted through emergency disposal practices during major outbreaks.

This reinterpretation reframes the Venetian quarantine system not as a passive repository of epidemic mortality, but as an active institutional process shaping which hosts, pathogens, and transmission chains entered the burial assemblage. Consequently, Lazzaretto Vecchio represents a unique long-term archive of infectious disease diversity preserved through nearly three centuries of quarantine practice.

## IMPLICATIONS FOR EVOLUTIONARY MEDICINE AND PATHOGEN HISTORY

The Venetian quarantine burial assemblage is distinct from other plague burial contexts in Europe. Unlike sites typically associated with single outbreaks dominated by plague [10, 11], the Venetian assemblage likely contains a broader diversity of pathogens. Under the dual-regime model (H2A–H2B), the apparent mismatch between recorded mortality and the surviving burial assemblage is not simply a problem of missing bodies, but evidence of selective demographic and pathogen filtering associated with quarantine practice. As a result, Lazzaretto Vecchio may preserve a unique three-century archive of intercepted infectious diseases, transmission failures, and disrupted epidemic chains.

**Figure 4:**
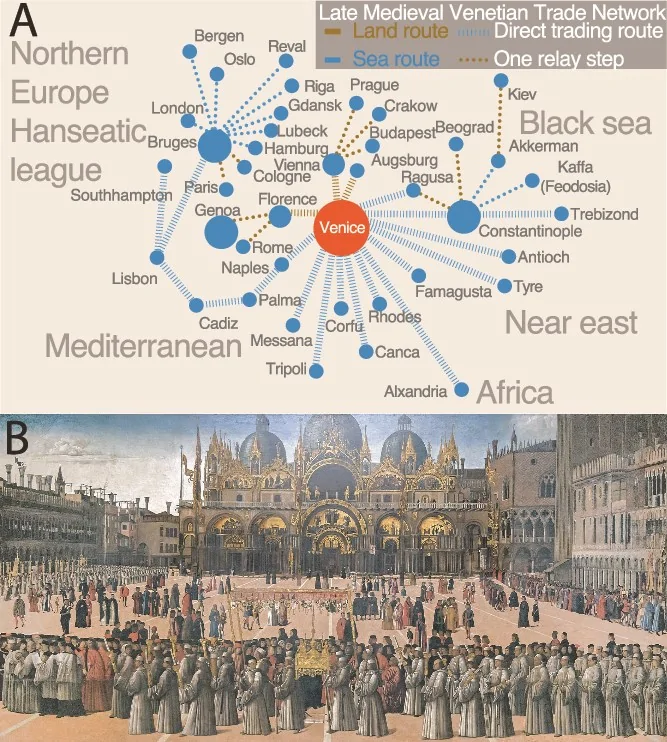
Venetian trade networks and the routes of contagion. (A) Reconstructed interaction network of major late-medieval maritime and overland trade centers based on Venetian archival data (adapted from Apellániz [18]). Nodes represent ports or commercial hubs, and edges denote documented trading routes and diplomatic contacts, illustrating the extensive mobility corridors linking Venice to Europe, North Africa, and the Eastern Mediterranean. This network serves as a proxy for historical pathways of pathogen movement and human genetic exchange. It provides a comparative framework for integrating genomic and isotopic data from individuals interred at *Lazzaretto Vecchio*, enabling alignment of mobility-driven exposure routes with host ancestry, pathogen diversity, and signatures of immune adaptation. (B) *Procession in Piazza San Marco* (1496) by Gentile Bellini depicts the cosmopolitan character of late-15th-century Venice, featuring Venetians, and diverse other groups active in the city. The scene reflects the demographic heterogeneity that accompanied Venice’s global maritime role and underscores both the city and its quarantine islands as biological and cultural bottlenecks where exposure, immunity, and contagion risk converged. Gentile Bellini (c. 1429–1507), *Procession in Piazza San Marco* (1496), tempera and oil on canvas, 367 × 745 cm, Gallerie dell’Accademia, Venice. Public-domain image via Wikimedia Commons.

**Figure 5:**
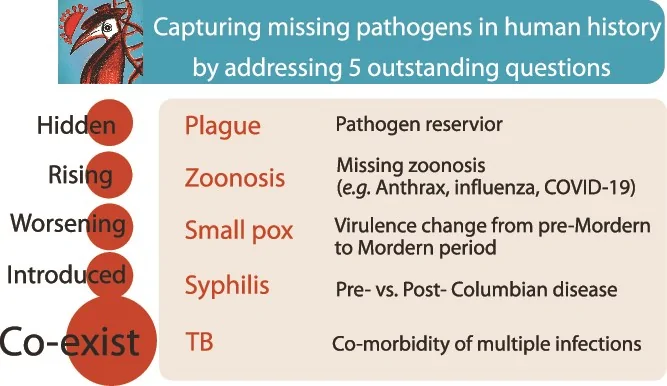
Five Key questions for recovering missing pathogens from human history. This conceptual framework highlights five outstanding questions in historical infectious-disease research that the study of *Lazzaretto Vecchio* is uniquely positioned to address. Representative pathogen examples are shown in red. Together, these questions define a roadmap for reconstructing the “shadow” history of infections suppressed or extinguished by early containment systems: (1) Hidden Disease Reservoirs: Where did pathogens such as *Yersinia pestis* persist between major outbreaks, and how did they re-emerge after long dormancy? (2) Undetected Zoonoses: Which animal-to-human infections, including anthrax and other rapidly fatal diseases, were intercepted at the quarantine frontier but never reached epidemic scale? (3) Virulence Shifts: How did containment, interrupted transmission, and selective bottlenecks influence transitions from mild to hyper-virulent forms, as seen in smallpox? (4) Pathogen Introductions: How can genomic data disentangle pre- and post-Columbian lineages, for example, in treponemal disease (*syphilis*)? (5) Co-Infection Dynamics: To what extent did multi-pathogen comorbidity, particularly with chronic infections such as *Mycobacterium tuberculosis*, shape mortality and immune vulnerability? Answering these questions requires an integrated analytical framework combining paleogenomics, proteomics, paleopathology, isotopic mobility data, and archaeological context, using the Venetian quarantine system as a uniquely time-structured containment archive for testing how policy, biology, and evolution intersected across centuries.

Because Venice functioned as a major maritime gateway linking Europe, Africa, and Asia (Fig. 4), the burial assemblage may contain a wide range of pathogens, including causal agents of plague, tuberculosis, salmonellosis, and other travel-associated infections [36–39]. This reinterpretation therefore extends beyond quarantine history and raises five broader questions in evolutionary medicine, pathogen ecology, and infectious disease evolution (Fig. 5).

Question 1. Where did epidemic pathogens go between outbreaks?

Historical plague dynamics [8, 9, 40–42] show long intervals between epidemic waves, yet pathogen persistence during these periods remains poorly understood. Individuals buried across multiple cycles at Lazzaretto Vecchio may preserve dormant lineages, aborted introductions, or failed transmission attempts, illuminating how pathogens persist, stall, or extinguish between outbreaks, e.g. the suspected accumulated pathogen diversity preserved in the lazaret may reflect dead-end lineages shaped by repeated bottlenecks.

Question 2. How many zoonotic threats never reached the city?

Most emerging infections originate in animals [43–45], yet many historical zoonoses left little trace. Because Venice intercepted travelers and cargo from Africa, the Near East, and Eurasia, the Lazzaretto may preserve pathogens halted at the maritime frontier, revealing unrecorded spillovers and missed zoonotic threats, before establishing sustained transmissions in human populations.

Question 3. What forces drive virulence shifts under containment?

Pathogens such as those for plague and smallpox exhibited major changes in virulence over time [46–51]. Venice’s long-term quarantine system provides an opportunity to test whether interrupted transmission, recurrent bottlenecks, and institutional barriers influenced the persistence, loss, or selection of virulent lineages.

Question 4. How did early globalization restructure pathogen diversity?

The post-1492 world reshaped global disease distributions [52–58]. As a hub linking Mediterranean, Asian, and African trade routes, Venice functioned as a pre-modern health checkpoint. Burials spanning pre- and post-Columbian exchange periods allow comparison of lineage replacement, persistence, and failed microbial migrations, under conditions shaped by early global connectivity and selective quarantine interception.

Question 5. How often did co-infections shape mortality?

Many infectious deaths result from synergistic co-infections rather than single agents [59–63]. Integrating both host and pathogen genetic data can reveal interacting pathogens and underlying host vulnerabilities, analogous to modern syndemics such as HIV–tuberculosis [62, 64, 65],. providing insight into how co-infections shape host–pathogen dynamics and mortality outcomes.

## CONCLUSION AND FUTURE PERSPECTIVES

Based on the current historical and archaeological evidence, we conclude that the discrepancy between the large documented death toll and the limited burial assemblage at Lazzaretto Vecchio cannot be adequately explained by the long-assumed resident-dominated burial model (the Venetian Plague House, H1). Instead, the available evidence is most consistent with a dual-regime system comprising traveler-dominated quarantine intake (the Ellis Island Effect, H2A) and crisis-phase diversion or cremation of local mortality (the Secret Republic Fire, H2B).

Together, this framework repositions Lazzaretto Vecchio as a controlled frontier archive of global infectious traffic: a rare longitudinal record of intercepted pathogens and an exceptional opportunity to investigate how quarantine systems shape pathogen transmission, diversity, and evolutionary dynamics.

The interpretations presented here remain incomplete and largely qualitative, highlighting the need for formal demographic, isotopic, and genetic investigations. Future work should develop a time-resolved quantitative framework integrating burial chronology, human mobility, kinship, and pathogen diversity.

By regulating who enters, survives, or is removed from transmission networks, quarantine systems impose selective pressures on pathogen transmission, persistence, and effective population size. In this sense, quarantine can be viewed as an institutional analogue of a population bottleneck, one whose biological consequences may be uniquely preserved within the burial assemblage of Lazzaretto Vecchio.

## Supplementary Material

Supplementary_materials_eoag016

## Data Availability

All data supporting the conclusions of this study are included within the manuscript and its Supplementary Material.
