# Supplementary material for: Quarantine as filter: a conceptual framework from early modern Venice
Source: Evol Med Public Health. 2026 Jul 19;14(1):1–12. doi: 10.1093/emph/eoag016 (PMC13489157; doi:10.1093/emph/eoag016)
Supplement: Supplementary file 1 [file supplementary_materials_eoag016.docx]

**Supplemental Text**

**Evidence of Historical Eyewitness Accounts: Rocco Benedetti (1576)**

Direct testimony from the Venetian chronicler Rocco Benedetti provides rare and explicit documentation of emergency plague management during the catastrophic outbreak of 1576 (*1*). His descriptions preserve both the procedural logic and sensory reality of operations at Lazzaretto Vecchio. We reproduce his eyewitness statements *verbatim* below, together with literal translations.

It is important to note that eyewitness accounts of Rocco Benedetti were written not in Latin or standardized Tuscan Italian, but in ancient Venetian, the administrative and civic language of the sovereign Republic of Venice (*1*). This linguistic choice is significant: Venetian functioned as a state language, used in government records, maritime law, medical directives, and civic reporting, rather than a regional dialect. Writing in Venetian signals that Benedetti’s testimony reflects an internal Venetian perspective, produced by individuals embedded within the social, political, and epidemiological infrastructure of the city rather than by external observers or later translators. The immediacy and idiomatic specificity of the original text, lexicon such as *Lazaretto Vecchio*, *brusciar*, and *spoglie da sacrificarsi a Vulcano*, capture local practices and attitudes that might otherwise be lost in standardized translation. Thus, the language of the record itself is a form of evidence: it situates these observations within Venice’s own civic narrative and institutional worldview, providing an authentic window into how plague control was experienced, understood, and morally justified by contemporaries.

Benedetti Quote #1 – Initial Response

Venetian (original):
“*Allhora i signori Proveditori della Sanità (...) terminarono per lo migliore di mandare quanto prima a Lazaretto Vecchio i feriti a risanarsi, et i sani che sotto un medesimo tetto vivevano con quelli al Lazaretto Novo a far contumacia quaranta giorni. Ordinarono poi col Senato che si brusciassero degli infetti tutte le robbe di casa*.”

Translation:
[The Health Office requested to send as soon as possible the injured (by the plague) to the Old Lazaret to recover, and the healthy that lived under the same roof to the New Lazaret to quarantine for forty days. They required along with the Senate that all things in the house belonging to the infected people be burnt].

Benedetti Quote #2 – Transport and Disposal

Venetian (original):
“*Ma molto più horribile spettacolo era la quantità delle barche che di continuo su e giù andavano, alcune a Lazareto Vechio cariche de feriti e di morti tutti mischiati insieme, altre a Lazareto Novo cariche di sani (...). Altre poi si vedevano andar fuori a certi luochi dessignati cariche di spoglie da sacrificarsi a Vulcano*.”

Translation:
[But more horrible was seeing the quantity of boats that traveled back and forth, some to the Old Lazaret full of injured and deceased all mixed, some to the New Lazaret full of healthy people (…). Other boats then could be seen going to certain locations decided by authorities full of bodies to sacrifice to Vulcan].

Benedetti Quote #3 – Burning on the Island

Venetian (original):
“*Ma lassando la città e volgendosi ai Lazaretti, dico in verità che dall’una parte il Lazaretto Vecchio rassembrava l’Inferno, ove da ogni lato usciva puzzore et insopportabil fettore, udivasi del continuo gemere et sospirare, et si vedevano da tutte le hore nuvoli di fumo stendersi in aere largamente per l’abrusciar de corpi*.”

Translation:
[But leaving the city aside and talking about the lazarets, I say in truth that the Old Lazaret resembled Hell, where from all sides bad smells and stench came out, there were sighs and cries coming from it, and at all times one could see clouds of smoke extending because of the burning of bodies].

Benedetti Quote #4 – Emergency Mass Cremation at Lido

Venentian (original):
“*Dove non si potendo più per la gran puzza abbrusciar i morti fu quindi poco discosto sul Lido, in luogo ditto alla Cavanella, fatto un campo santo, nel quale fatte profondissime cave quivi si ponevano, mettendosi segondo si faceva al detto Lazaretto, una mano de corpi, una di calcina viva et una di terra, et così di mano in mano fino che ne potevano stare, in modo che da un giorno all’altro erano tutti incineriti.”*

Translation:
[When it was no longer possible to burn the bodies because of the smells, in the island of Lido, at the location of Cavanella, a cemetery was erected, where inside deep trenches were laid down bodies to burn, layered as bodies, followed by live lime and soil, and stacked as many times as they could, so that from one day to the other they were all burnt].

Taken together, Benedetti’s eyewitness narrative indicates that bodies from the city were not buried but burned, and that mass cremation replaced burial during crisis conditions. While a small number of infected individuals may have survived quarantine, the majority, *whether still alive or already dead*, were burned immediately upon arrival, consistent with his repeated description of “injured and dead mixed” on transport boats.

It was not the first time that Venice diverged from mainstream Christian expectations. The Venetian Republic maintained a long-standing independence from papal authority, and tensions with the Roman Church over jurisdiction and ritual practice are well documented. This political and cultural autonomy helps explain why emergency mass cremation could be implemented in Venice during epidemic crises, even though such practices would have been unacceptable in most other European settings. As an illustration of Venice’s distinctive relationship to religious symbolism, the winged lion of St. Mark, the central emblem of the Republic, has been recently shown to derive from a reworked pagan figure (*2*), demonstrating the city’s history of pragmatic adaptation rather than strict religious conformity.

References:

1.	R. Benedetti, D. Calabi, L. Molà, S. Rauch, E. Svalduz, *Venezia 1576, la peste: una drammatica cronaca del Cinquecento*. (Cierre Edizioni, Sommacampagna (VR), 2021).

2.	G. Artioli *et al.*, The Chinese identity of St Mark’s bronze ‘Lion’ and its place in the history of medieval Venice. *Antiquity* **99**, 1372-1388 (2025).
